# Author Correction: Manipulating polariton condensates by Rashba-Dresselhaus coupling at room temperature

**DOI:** 10.1038/s41467-022-32032-6

**Published:** 2022-07-25

**Authors:** Yao Li, Xuekai Ma, Xiaokun Zhai, Meini Gao, Haitao Dai, Stefan Schumacher, Tingge Gao

**Affiliations:** 1 Department of Physics, School of Science, Tianjin University, Tianjin, 300072 China; 2 Tianjin Key Laboratory of Molecular Optoelectronic Science, Institute of Molecular Plus, Tianjin University, Tianjin, 300072 China; 3 Department of Physics and Center for Optoelectronics and Photonics Paderborn (CeOPP), Universität Paderborn, Warburger Strasse 100, 33098 Paderborn, Germany; 4 Tianjin Key Laboratory of Low Dimensional Materials Physics and Preparing Technology, School of Science, Tianjin University, Tianjin, 300072 China; 5 Wyant College of Optical Sciences, University of Arizona, Tucson, AZ 85721 USA

**Keywords:** Quantum fluids and solids, Polaritons

Correction to: *Nature Communications* 10.1038/s41467-022-31529-4, published online 01 July 2022

The original version of this Article contained an error in Ref. 18, which was incorrectly given with the wrong page number as: *Nat. Commun.* **8**, 1–6 (2017). The correct form of Ref. 18 is: *Nat. Commun.* **8**, 14540 (2017).

The original version of this Article contained an error in Ref. 22, which was incorrectly given with the wrong page number as: *Nat. Commun.* **7**, 1–6 (2016). The correct form of Ref. 22 is: *Nat. Commun.* **7**, 10983 (2016).

The original version of this Article contained an error in Ref. 33, which was incorrectly given with the wrong page number as: *Light Sci. Appl.* **7**, 1–6 (2018). The correct form of Ref. 33 is: *Light Sci. Appl.* **7**, 74 (2018).

The original version of this Article contained an error in Ref. 40, which was incorrectly given with the wrong page number as: *Nat. Commun.* **11**, 1–7 (2020). The correct form of Ref. 40 is: *Nat. Commun.* **11**, 897 (2020).

This has been corrected in the PDF version of the Article.

