# Supplementary material for: Manipulating polariton condensates by Rashba-Dresselhaus coupling at room temperature
Source: Nat Commun. 2022 Jul 1;13:3785. doi: 10.1038/s41467-022-31529-4 (PMC9249758; doi:10.1038/s41467-022-31529-4)
Supplement: Supplementary file 1 — Supplementary Information [file 41467_2022_31529_MOESM1_ESM.pdf]

Supplementary Information: Manipulating polariton condensates by Rashba-Dresselhaus coupling at room temperature

Yao Li,[1,2] Xuekai Ma,[3,*] Xiaokun Zhai,[1,2] Meini Gao,[4] Haitao Dai,[4] Stefan Schumacher,[3,5] and Tingge Gao[1,2,†]

[1]*Department of Physics, School of Science, Tianjin University, Tianjin 300072, China*
[2]*Tianjin Key Laboratory of Molecular Optoelectronic Science, Institute of Molecular Plus, Tianjin University, Tianjin 300072, China*
[3]*Department of Physics and Center for Optoelectronics and Photonics Paderborn (CeOPP), Universität Paderborn, Warburger Strasse 100, 33098 Paderborn, Germany*
[4]*Tianjin Key Laboratory of Low Dimensional Materials Physics and Preparing Technology, School of Science, Tianjin University, Tianjin 300072, China*
[5]*Wyant College of Optical Sciences, University of Arizona, Tucson, AZ 85721, USA*

Supplementary Note 1: Design of the microcavity

We design the microcavity by calculating both the reflectivity and the electric field distribution by using the transfer matrix method. Supplementary Figure 1(a) is the reflectivity of the microcavity. Supplementary Figure 1(b) shows that the perovskite is placed at the local maximum of the electric field distribution of the microcavity. Although the perovskite microplate is placed at the edge of the cavity layer, however, the giant exciton binding energy and oscillator strength can sustain strong coupling between the excitons and cavity photon modes.

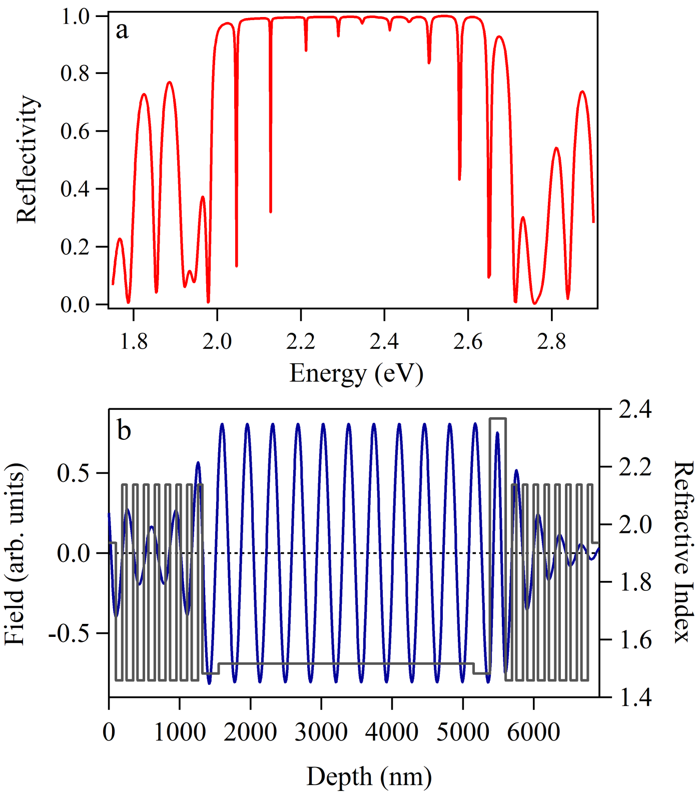

Supplementary Figure 1. The liquid crystal microcavity (a) Simulated reflectivity of the liquid crystal microcavity. (b) Simulated electric field distribution of the liquid crystal microcavity at 2.2892 eV.

* *Corresponding author; xuekai.ma@gmail.com
† *Corresponding author; tinggegao@tju.edu.cn

Supplementary Note 2: Momentum-space spectroscopy setup

To explore the angle resolved momentum information of the exciton polaritons in the microcavity, the angle-resolved microphotoluminescence measurement was carried out at room temperature by our home-built spectroscopy setup, as shown in Supplementary Figure 2. The microcavity is excited by an expanded femtosecond laser (400 nm) which is focused onto the sample with the spot size of around 58 μm. The photons emitted by the microcavity are collected by the same objective and sent to the spectrometer. The last lens closest to the spectrometer can be flipped to observe the real space imaging of the polaritons.

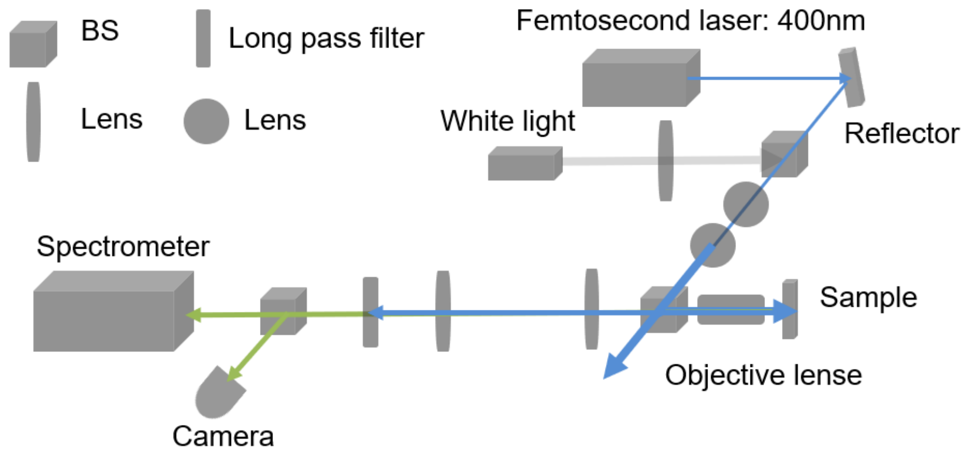

Supplementary Figure 2. Schematic of the angle-resolved microphotoluminescence setup.

Supplementary Note 3: Dispersion of the liquid crystal microcavity below and above the threshold

We re-plot the dispersion in Fig. 2(b) in log scale and place it with Fig. 2(a) together. It shows that above the threshold the dispersion of the microcavity at larger momentum is the same as that below the threshold (Supplementary Figure 3(a)), confirming the polariton condensation in the strong coupling regime. A much clearer evidence of the strong coupling can be found in Supplementary Figure 3(c).

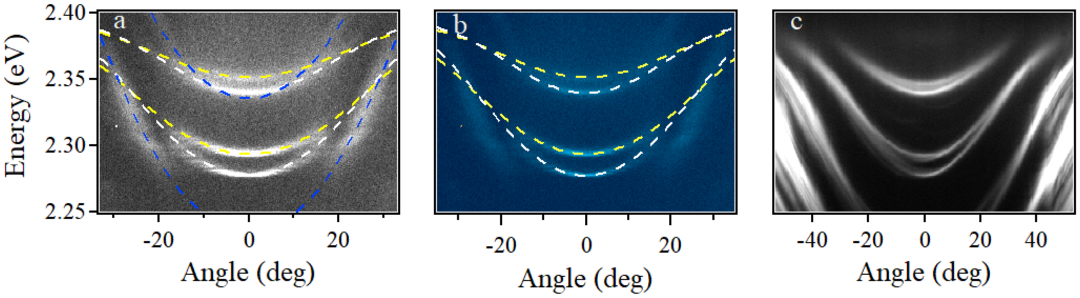

Supplementary Figure 3. Polariton condensation in the liquid crystal microcavity. Dispersion of the liquid crystal microcavity (a) below and (b) above (log scale) the threshold.The detuning of LP1, LP2, LP3, and LP4 are -17.5 meV, -36 meV, -94.5 meV, and -113 meV respectively. (c) Dispersion at much larger angles of the microcavity below the threshold, measured by using a larger-NA objective.

Supplementary Note 4: Hopfield coefficients

The exciton and cavity photon components of each LP branch are calculated and shown in Supplementary Figure 4. With decreasing the detuning between the cavity photons and excitons (more negative), the photon fraction gradually increases from LP1 to LP4, while the exciton fraction gradually decreases from LP1 to LP4 when the cavity photon modes are away from the exciton resonance.

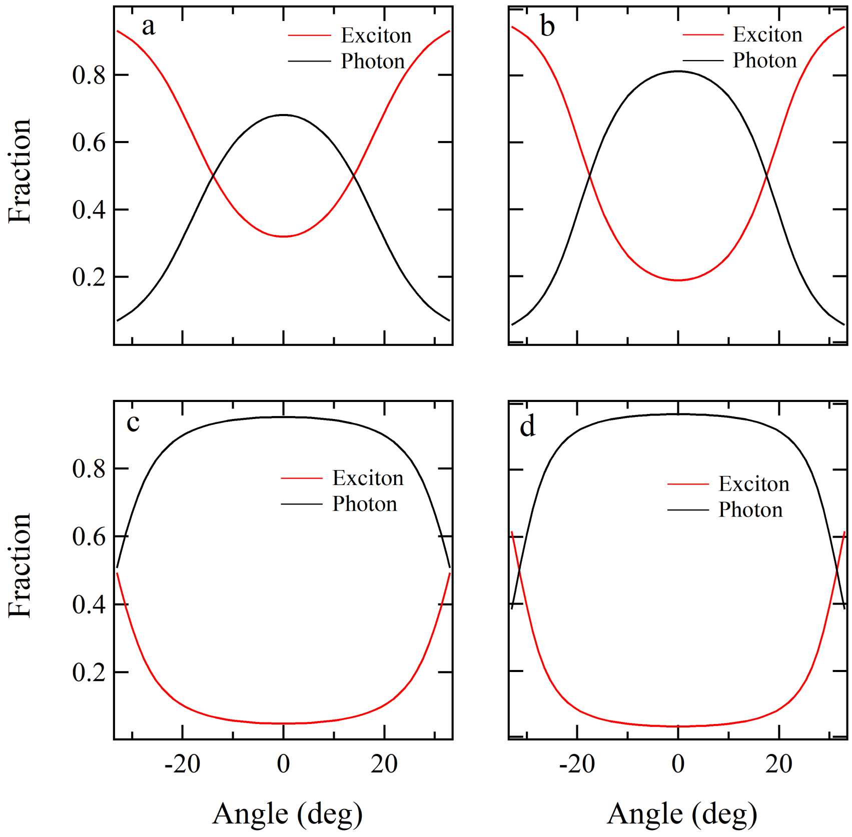

Supplementary Figure 4. Fraction of exciton and photon contribution for LP1(a), LP2(b), LP3(c), and LP4(d) respectively.

Supplementary Note 5: Interference of the condensates

Here the macroscopic coherence built above the threshold is measured. In the experiments, we rotate the real space imaging of the polariton condensate by 180 degrees. Clear interference fringes are observed as shown in Supplementary Figure 5(c). On the contrary, one cannot see any fringes below the threshold (Supplementary Figure 5(f)), thus the macroscopic coherence accompanying the polariton condensation is confirmed.

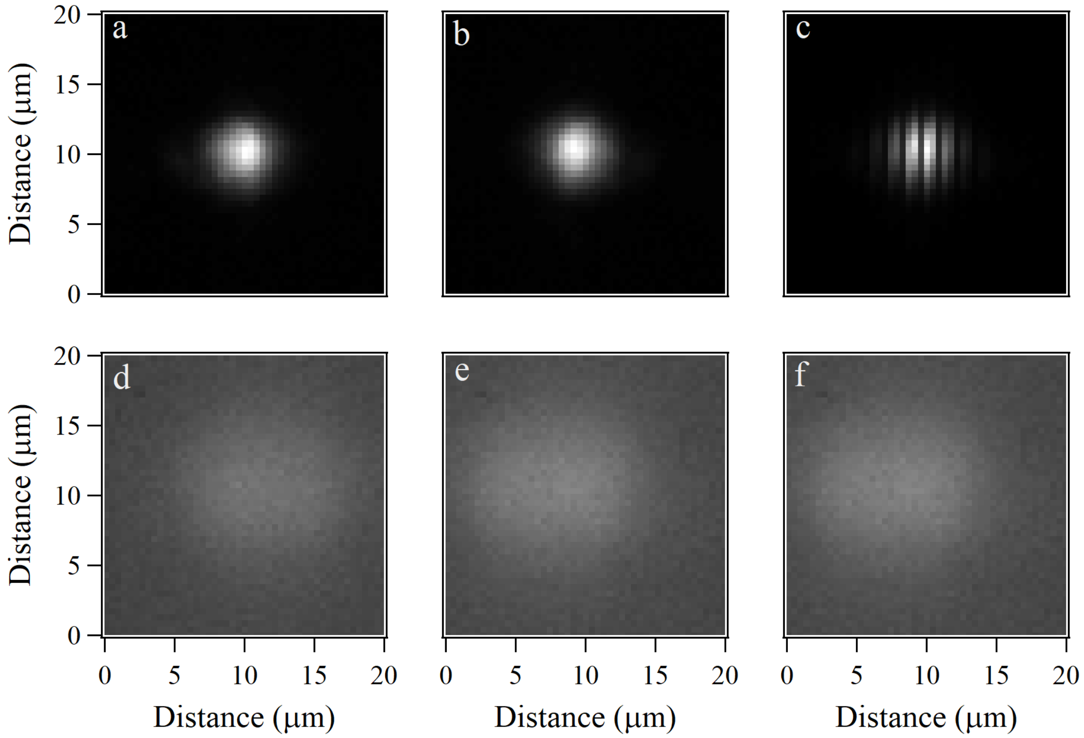

Supplementary Figure 5. Interference of the condensates. Real space photoluminescence image measured (a) above and (d) below the threshold from one arm of the Michelson interferometer. Real space photoluminescence image obtained (b) above and (e) below the threshold from the second arm by using a retroreflector to flip the image. Interference pattern after superposition of the images (c) above and (f) below the threshold from the two arms of the Michelson interferometer.

Supplementary Note 6: Dispersion at different pump fluence

In this section we plot the dispersion curves of the microcavity at 18.4 μJ/cm^2 and 20.7 μJ/cm^2 when the voltage is 2 V. Initially the polariton condensation occurs at LP4 branch as shown in the main text. Increasing the pump fluence leads to that the polaritons can condense at both LP3 and LP4 branches.

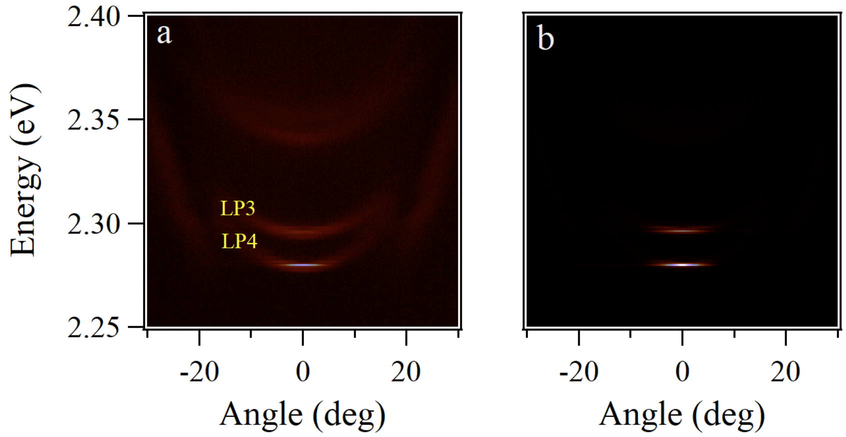

Supplementary Figure 6. The dispersion at pump fluence (a) 18.4 μJ/cm^2 and (b) 20.7 μJ/cm^2.

Supplementary Note 7: Polariton dispersion and condensation at different voltages

In this section we plot the dispersion of the microcavity at different voltages, below and above the threshold. As we can see that the two branch polaritons approach each other when the voltage is increased from 3 V to 4.5 V below the condensation threshold. At the voltage of 4.5 V, the dispersion is shifted by $k_x = \pm 0.258\ \mu\text{m}^{-1}$, as discussed in the main text. The same process occurs also above the threshold.

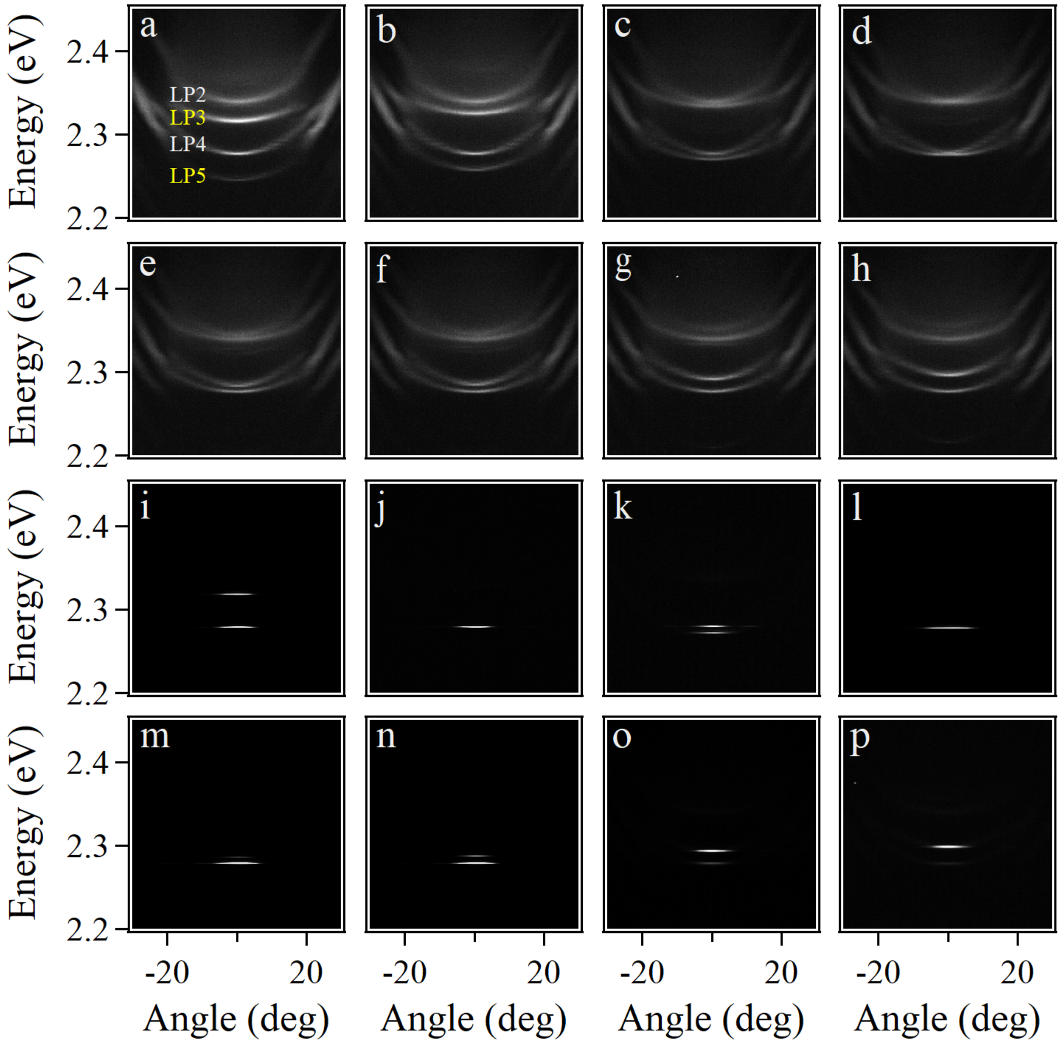

Supplementary Figure 7. Modulation of the voltage on polariton dispersion and condensation. The dispersion of the microcavity below (a-h) and above (i-p) the threshold as a function of the voltage. The voltage for (a-h) and (i-p) are 3.0 V, 3.5 V, 4.1 V, 4.5 V, 4.9 V, 5.0 V, 5.5 V, 6.0 V,respectively.

Supplementary Note 8: Stokes parameters in k-space

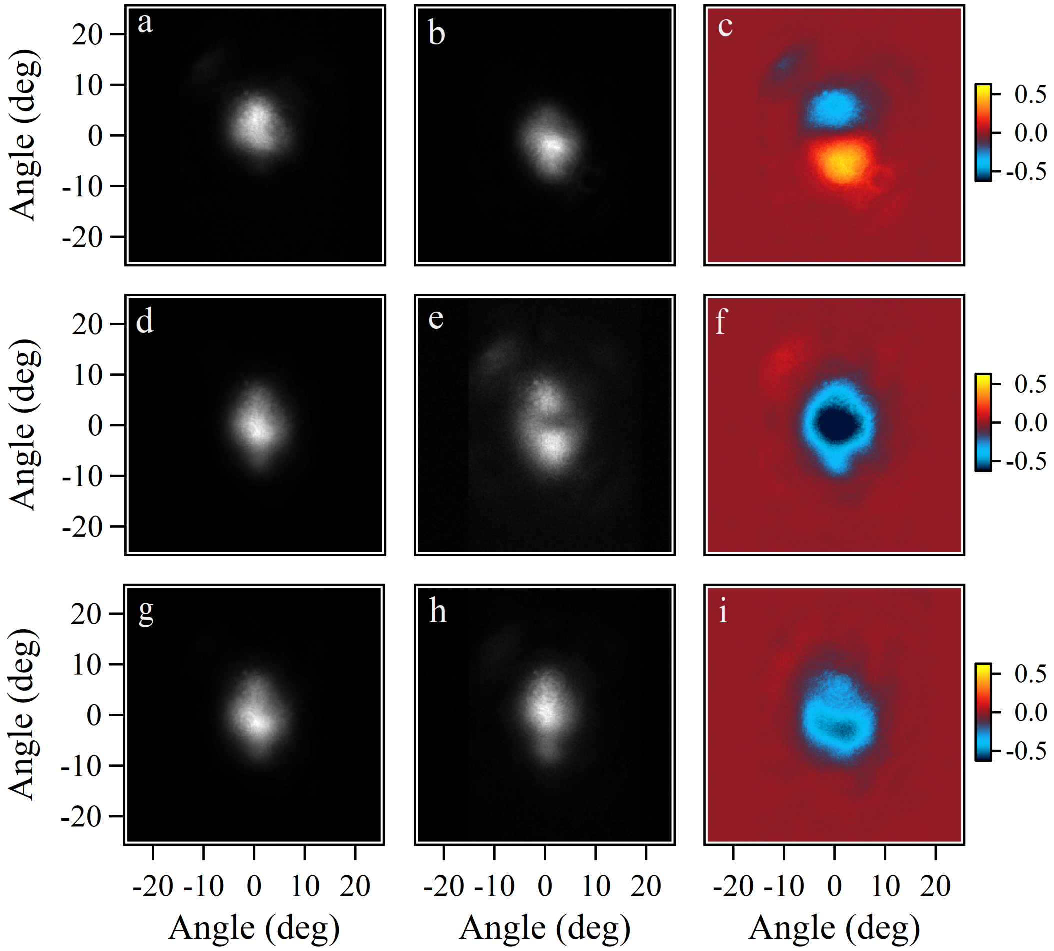

Supplementary Figure 8. Stokes parameters in k-space (a, b) Left- and right-hand circularly polarized momentum space images, (d, e) Horizontally and vertically linearly polarized momentum space images, (g, h) Diagonally and antidiagonally linearly polarized images of the polariton condensate tuned by the RD effect. (c, f, i) are the polarized images corresponding to (a, b), (d, e), and (g, h), respectively.

Supplementary Note 9: Stokes parameters in real space

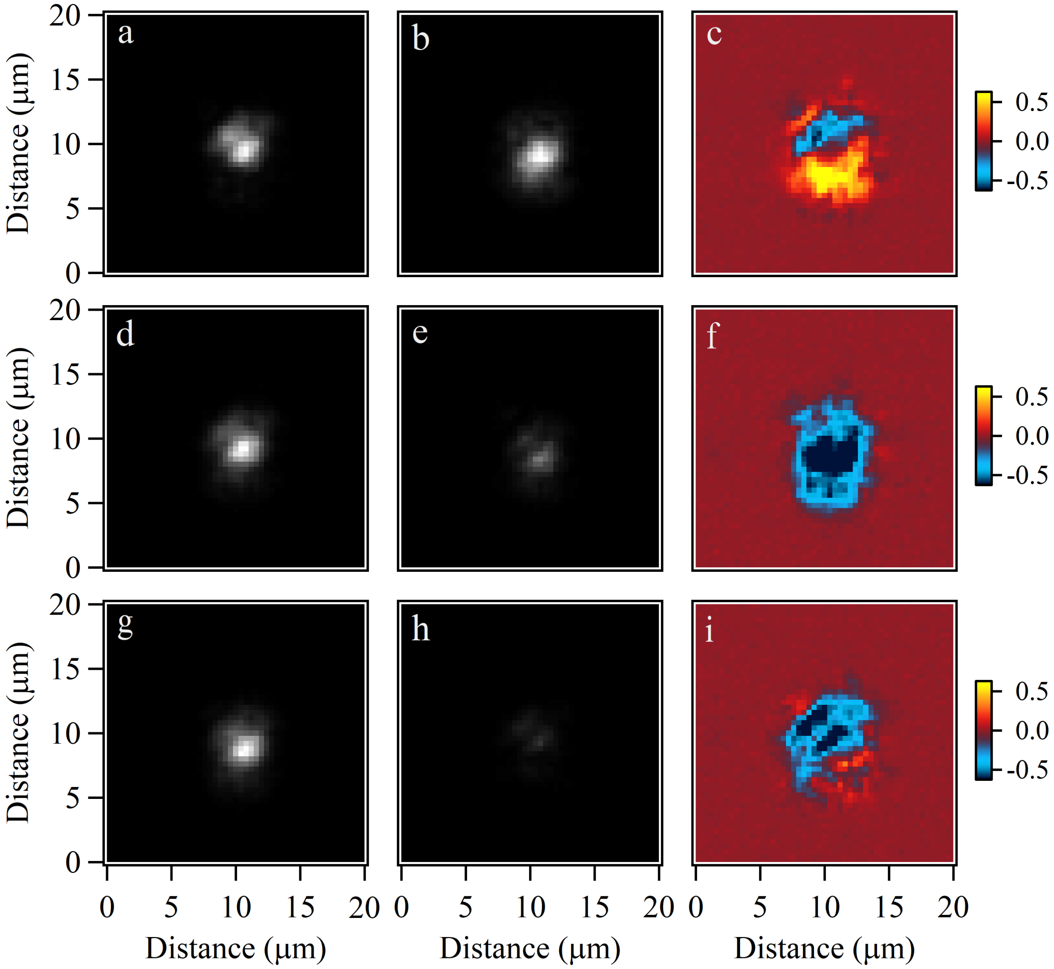

Supplementary Figure 9. Stokes parameters in real space (a, b) Left- and right-hand circularly polarized real space images, (d, e) Horizontally and vertically linearly polarized real space images, (g, h) Diagonally and antidiagonally linearly polarized images of the polariton condensate tuned by the RD effect. (c, f, i) are the polarized images corresponding to (a, b), (d, e), and (g, h), respectively.

Supplementary Note 10: The influence of RD effect on the discrete energy levels

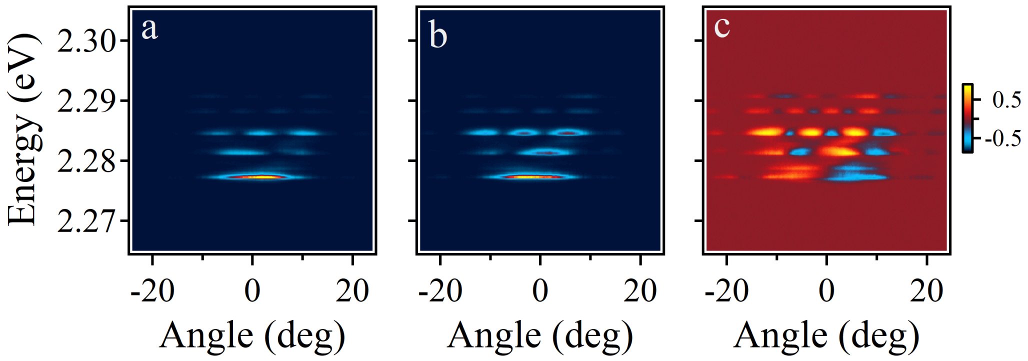

Supplementary Figure 10. Rashba-Dresselhaus effect for discrete polariton condensates. (a) Left- and (b) right-hand circularly polarized dispersion above the threshold at 4.5 V. (c) Circularly polarized dispersion corresponding to (a, b).
